# Spontaneous spinal epidural hematoma leading to acute paraplegia: a case report

**DOI:** 10.1186/s13256-023-04297-y

**Published:** 2023-12-14

**Authors:** Yu Liu, Rajeesh George, Gamaliel Yu Heng Tan

**Affiliations:** Department of Orthopaedic Surgery, Ng Teng Fong General Hospital, Singapore, Singapore

**Keywords:** Spinal hematoma, Spontaneous, Decompression, Paraplegia, Case report

## Abstract

**Background:**

Spontaneous spinal epidural hematoma is an infrequent yet potentially debilitating condition characterized by blood accumulation in the epidural space, with only 300 documented cases globally. Although the exact etiology of spontaneous spinal epidural hematoma remains poorly understood, theories suggest arteriovenous malformations, rupture of epidural vessels, or epidural veins as possible causes.

**Case presentation:**

This study presents a 58-year-old Malay woman patient from Singapore with well-controlled hypertension, hyperlipidemia, type II diabetes mellitus, and microscopic hematuria. Despite a prior cystoscopy revealing no abnormalities, she presented to the emergency department with sudden-onset back pain, weakness, and numbness in both lower limbs. Rapidly progressing symptoms prompted imaging, leading to the diagnosis of a spinal epidural hematoma from thoracic (T) 9 to lumbar (L) 1. Prompt decompressive surgery was performed, and the patient is currently undergoing postoperative rehabilitation for paralysis.

**Conclusion:**

This case emphasizes the severity and life-altering consequences of spontaneous spinal epidural hematomas. Despite various proposed causative factors, a definitive consensus remains elusive in current literature. Consequently, maintaining a low threshold of suspicion for patients with similar presentations is crucial. The findings underscore the urgent need for swift evaluation and surgical intervention in cases of acute paraplegia.

## Background

Spontaneous spinal epidural hematoma (SSEH) is a rare but potentially debilitating condition characterized by the accumulation of blood in the epidural space, with only 300 cases reported worldwide. The exact etiology of SSEH is still poorly known but has been postulated to be due to arteriovenous malformations, rupture of epidural vessels, or epidural veins.

## Case presentation

Our patient is a 58-year-old Malay woman with a previous medical history of well-controlled hypertension, hyperlipidemia, type II diabetes mellitus, and microscopic hematuria. She had undergone a cystoscope for evaluation of the microscopic hematuria in which there were no abnormalities detected. There was no history of any recent infection, trauma, coagulopathy, or recent surgery. She presented to our emergency department with sudden-onset back pain, associated with weakness and numbness in both lower limbs that developed later. The symptoms began abruptly and had progressed rapidly over a few hours. The initial blood investigations revealed the full blood count, electrolyte panel, international normalized ratio (INR), prothrombin time (PT), and activated partial thromboplastin time (aPTT) to be within normal limits. She was later worked up with imaging and diagnosed with a spinal epidural hematoma, in which prompt decompressive surgery was done from thoracic (T) 9 to lumbar (L) 1. She is still undergoing rehabilitation postoperatively for her paralysis.

## Discussion and conclusion

This presented case serves as a reminder of the potential severity and life-altering consequences of spontaneous spinal epidural hematomas. While there is a spectrum of proposed causative factors, a definitive consensus remains elusive within the current literature. Therefore, a low threshold of suspicion should be maintained when encountering patients with such presentations. This case underscores the urgent need for rapid evaluation and surgical intervention when acute paraplegia manifests.

### Timeline of events


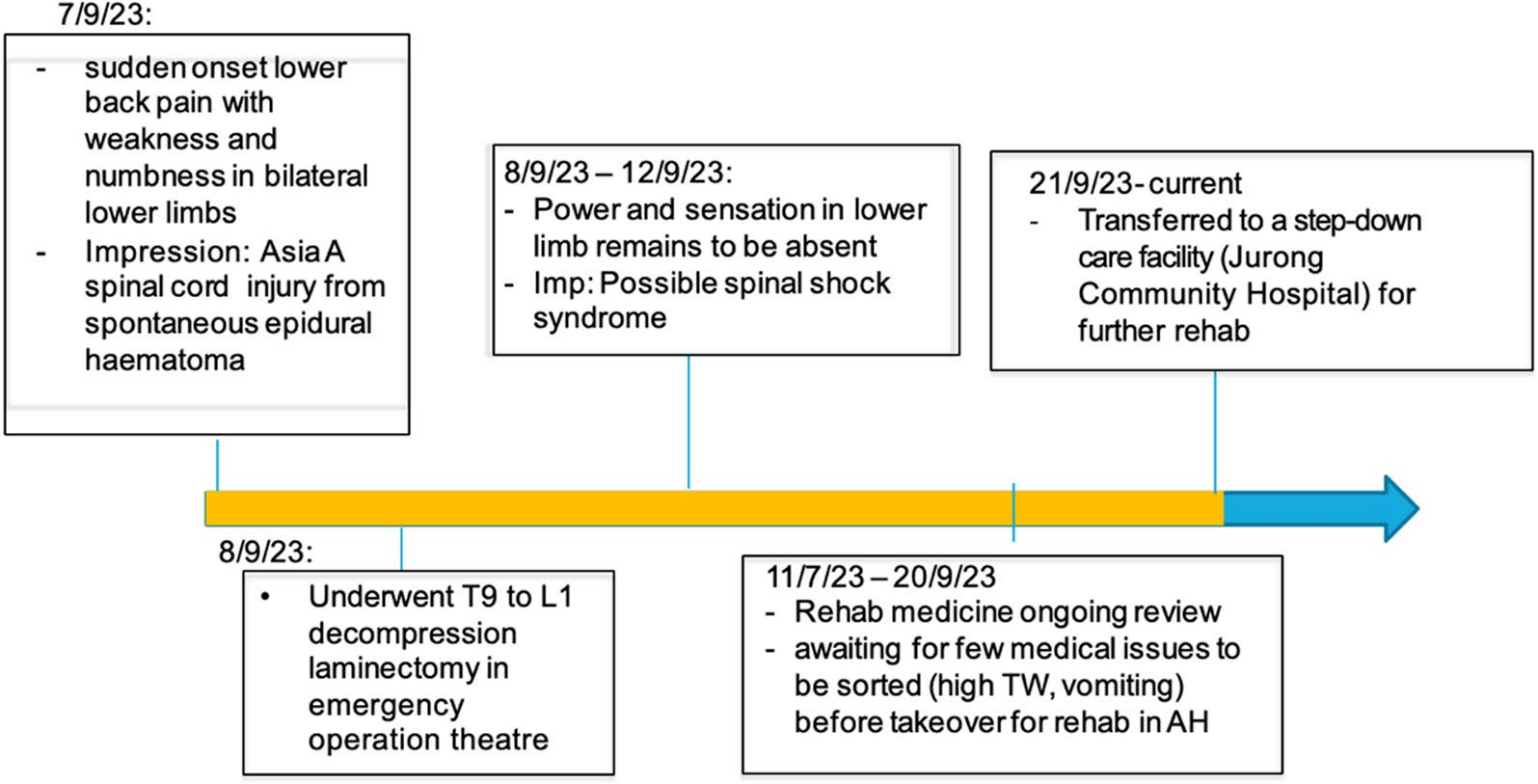


### Clinical findings

Upon examination, the patient exhibited flaccid paraplegia with a sensory level at T12. Deep tendon reflexes in the lower limbs were diminished, and the Babinski sign was absent bilaterally. There were no signs of upper limb weakness or cranial nerve involvement. Laboratory tests, including a complete blood count and coagulation profile, were within normal limits. The patient was also noted to have hypertensive urgency, with a systolic blood pressure of 188 mmHg, which came down after administration of anti-hypertensives.

### Imaging

**Fig. 1:**
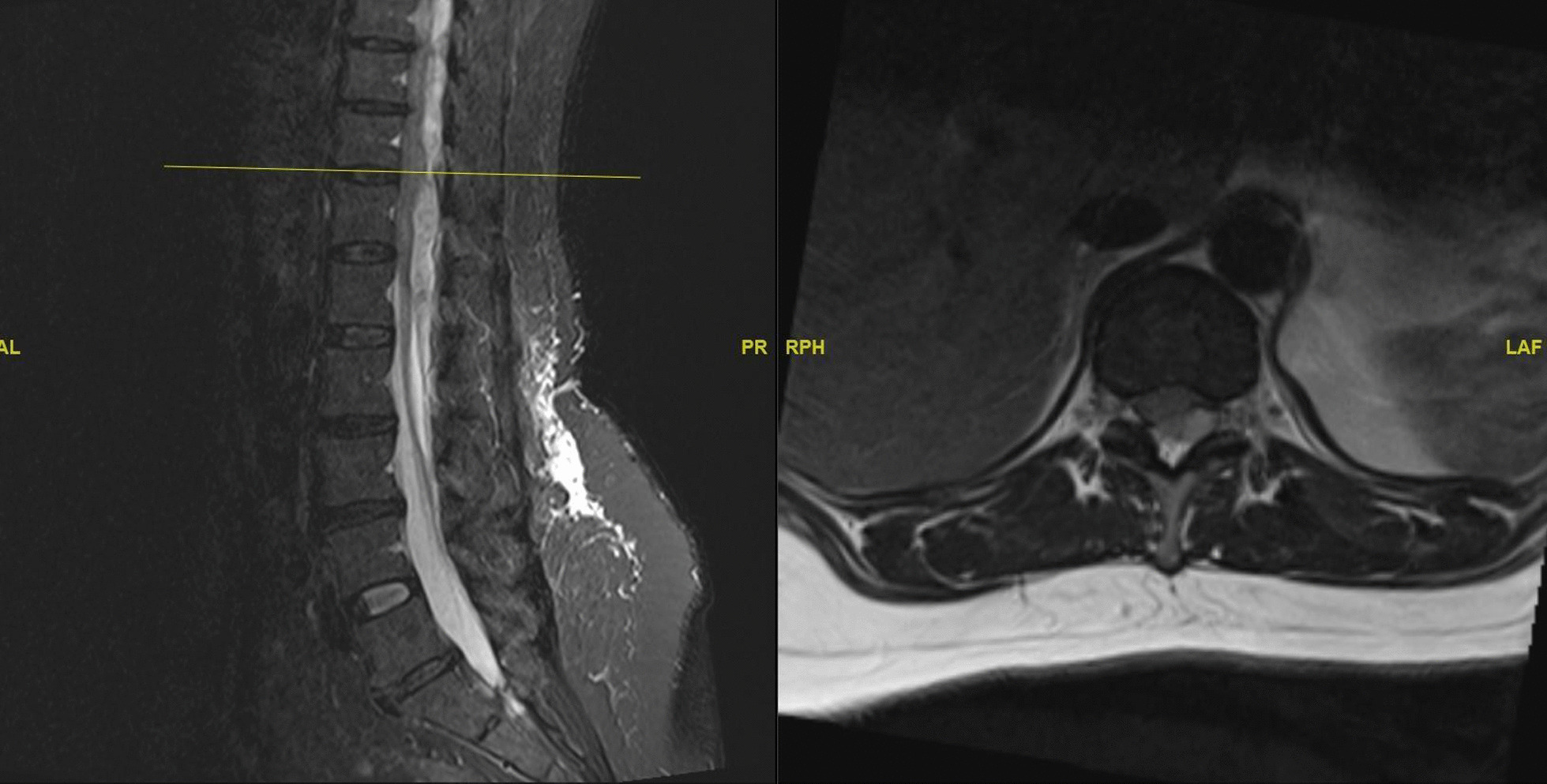
Pre-operative MRI whole spine

Initial magnetic resonance imaging (MRI) of the whole spine (Fig. 1) revealed a spinal epidural hematoma extending from the T9 to L1 levels, causing significant spinal cord compression.

### Treatment and management

The patient was reviewed by the on-call team and consultant-in-charge in the emergency department. The above MRI images were completed and reviewed within the next hour.

Due to the rapidly deteriorating neurological status, immediate surgery was deemed necessary. The patient received a first dose of intravenous dexamethasone 8 mg and 4 mg every 8 hours and underwent laminectomy of the T9 to L1 vertebrae within 3 hours of MRI completion to alleviate spinal cord compression and evacuate the hematoma.

Intraoperatively, a sizable epidural hematoma was identified and successfully removed. Baseline somatosensory evoked potentials (SSEP) in the bilateral upper limb were strong but weak in bilateral lower limbs, with no change intraoperatively. Baseline motor evoked potential (MEP) in the upper limb was good but there was no response in bilateral lower limb, with no change intraoperatively. Baseline electromyography (EMG) in bilateral lower limbs had on-and-off irritation.

SSEP in bilateral lower limbs improved following the operation. However, it is noteworthy that the patient’s mean arterial pressure (MAP) occasionally dropped below 50 mmHg before the surgery commenced.

Postoperatively, the patient underwent rehabilitation in a step-down facility. Her motor and sensory function remained absent.

Discussion was made with patient and department of radiology about the utility in performing a computed tomography (CT) whole spine angiogram to rule out an arteriovenous malformation. However, due to low yield and high radiation, patient decided against it.

**Fig. 2:**
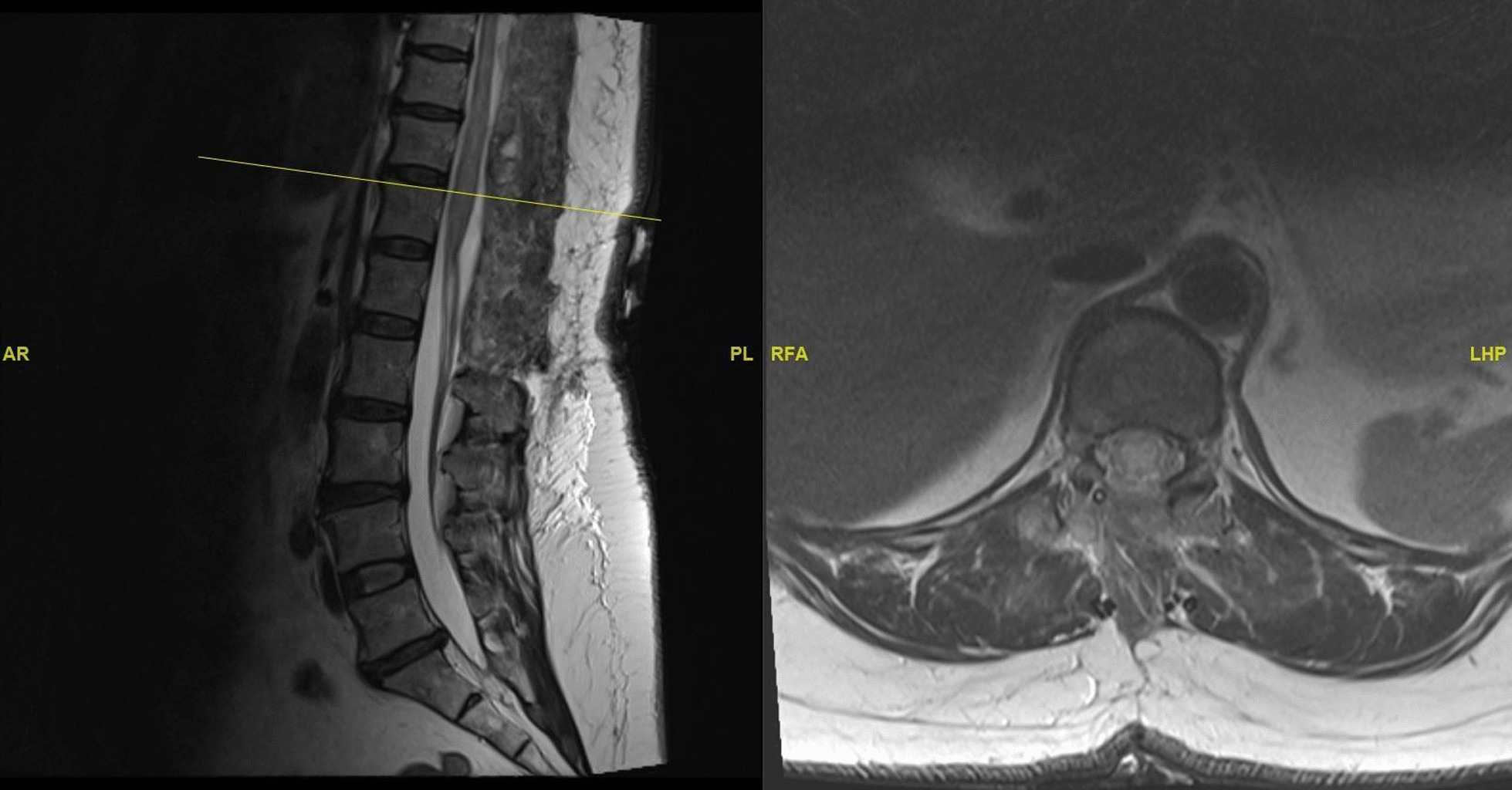
Post-operative MRI whole spine

Repeat MRI whole spine (Fig. 2) shows resolution of the hematoma at the affected levels.

### Discussion

Spontaneous spinal epidural hematomas are exceptionally rare occurrences, predominantly associated with vascular anomalies or coagulation disorders. This case underscores the paramount significance of prompt diagnosis and timely surgical intervention in cases of acute, severe spinal cord compression [1]. Clinical presentations encompass a wide array of symptoms, including motor weakness, sensory deficits, bowel and bladder dysfunction, neck pain, back pain, leg pain, abdominal discomfort, and gait instability [2]. Considerations should also be made for other differentials such as epidural abscess, spinal cord ischemia, and transverse myelitis [3].

Magnetic resonance imaging (MRI) remains the gold standard for diagnosing such conditions, providing precise visualization of the hematoma and its impact on the spinal cord [4]. A thorough literature review reveals that early surgical decompression, as exemplified in this case, particularly in patients with incomplete neurological deficits [5] or the absence of sensory deficits [6], is closely associated with superior neurological outcomes [1, 5].

It is imperative to emphasize that vigilant efforts must be made to maintain mean arterial pressure (MAP) above 80 mmHg, as maintaining adequate perfusion pressure is crucial in preventing secondary spinal cord injury [7, 8].

## Data Availability

Not applicable.

## Abbreviations

aPTT: Activated partial thromboplastin time
CT: Computed tomography
EMG: Electromyography
INR: International normalized ratio
MRI: Magnetic resonance imaging
MAP: Mean arterial pressure
MEP: Motor evoked potential
PT: Prothrombin time
SSEH: Spontaneous spinal epidural hematoma
SSEP: Somatosensory evoked potentials

## References

[1] Dziedzic T, Kunert P, Krych P, Marchel A (2015). Management and neurological outcome of spontaneous spinal epidural hematoma. J Clin Neurosci.

[2] Babayev R, Ekşi MŞ (2016). Spontaneous thoracic epidural hematoma: a case report and literature review. Childs Nerv Syst.

[3] Baek BS, Hur JW, Kwon KY, Lee HK (2008). Spontaneous spinal epidural hematoma. J Korean Neurosurg Soc..

[4] Al-Mutair A, Bednar DA (2010). Spinal epidural hematoma. J Am Acad Orthop Surg.

[5] Zhong W, Chen H, You C, Li J, Liu Y, Huang S (2011). Spontaneous spinal epidural hematoma. J Clin Neurosci.

[6] Figueroa J, DeVine JG (2017). Spontaneous spinal epidural hematoma: literature review. J Spine Surg.

[7] Gaudin XP, Wochna JC, Wolff TW, Pugh SM, Pandya UB, Spalding MC, Narayan KK (2019). Incidence of intraoperative hypotension in acute traumatic spinal cord injury and associated factors. J Neurosurg Spine.

[8] Hawryluk G, Whetstone W, Saigal R, Ferguson A, Talbott J, Bresnahan J, Dhall S, Pan J, Beattie M, Manley G (2015). Mean arterial blood pressure correlates with neurological recovery after human spinal cord injury: analysis of high frequency physiologic data. J Neurotrauma..

